# Paliperidone Palmitate Toxicity in Chronic Kidney Disease: A Case of Life-Threatening Autonomic Failure

**DOI:** 10.7759/cureus.92350

**Published:** 2025-09-15

**Authors:** Rishu Rishu, Amr Abdallah, Darshan Pandit

**Affiliations:** 1 General Internal Medicine, Russells Hall Hospital, The Dudley Group NHS Foundation Trust, Dudley, GBR; 2 Gastroenterology, Russells Hall Hospital, The Dudley Group NHS Foundation Trust, Dudley, GBR; 3 Critical Care, Russells Hall Hospital, The Dudley Group NHS Foundation Trust, Dudley, GBR

**Keywords:** atypical antipsychotics toxicity, autonomic disturbance, autonomic involvement, chronic kindey disease, paliperidone palmitate, schizophrenia and other psychotic disorders

## Abstract

Long-acting injectable antipsychotics (LAIs) are among the treatment options available for patients with schizophrenia. This case report discusses a case of a 50-year-old patient with a complex medical background, including chronic kidney disease (CKD), who presented clinically unwell with autonomic instability requiring cardio-respiratory support. The patient was on three-monthly doses of long-acting injectable antipsychotics; the last dose was administered two months ago before the presentation. The patient’s creatinine clearance (CrCl) at the time of admission was 12 ml/min, and the estimated glomerular filtration rate (eGFR) was 9 ml/min/1.73 m^2^. This case highlights the autonomic side effects of long-acting injectable antipsychotics (LAIs) in a patient with chronic kidney disease (CKD), underscoring the need for close therapeutic drug monitoring and consideration of alternative treatment approaches in patients with moderate to severe renal dysfunction due to the risk of drug accumulation and toxicity.

## Introduction

Poor adherence to pharmacological treatment remains a major challenge in managing patients with schizophrenia [1,2]. Fewer than 65% of patients maintain full adherence after just a few weeks, and this figure declines to merely 25% over two years [1,3]. Long-acting injectable antipsychotics (LAIs) were introduced in the 1960s to specifically tackle the issue of nonadherence [4]. One such LAI is paliperidone palmitate. Paliperidone (9-hydroxy-risperidone) is the primary active metabolite of risperidone [1,2,4,5]. 

Paliperidone palmitate primarily acts as an antagonist at central D2 dopamine and 5-HT2A serotonin receptors. Additionally, it blocks alpha-1 and alpha-2 adrenergic receptors, as well as H1 histamine receptors [1,2,4,6]. PP1M (paliperidone palmitate once-monthly) was introduced as a long-acting injectable administered as a single dose every 4 weeks, without the need for oral supplementation. PP3M (paliperidone palmitate three-monthly) was subsequently developed for patients who have been stabilised on PP1M for at least 4 months, offering a longer dosing interval [1,7]. Paliperidone is primarily eliminated through renal excretion. Therefore, its use is not recommended in patients with moderate to severe renal impairment [4,8].

We present a case of a 50-year-old chronic kidney disease (CKD) patient who presented with autonomic instability due to potential Paliperidone palmitate accumulation pertaining to decreased renal clearance.

## Case presentation

We report the case of a 50-year-old patient with a complex medical background with chronic kidney disease stage 5, type 2 diabetes mellitus (T2DM) with established diabetic retinopathy, hypertension, obstructive sleep apnoea, prior lacunar infarct, and schizophrenia, which was managed with LAIs, aspirin, atorvastatin, furosemide, hydralazine, ticagrelor, insulin glargine, procyclidine, and darbepoetin. The stage 5 chronic kidney disease (CKD) was managed with fluid restriction while awaiting formation of an arteriovenous (AV) fistula for haemodialysis. 

The patient presented with acute hemodynamic compromise and required intensive multi-organ support. Before hospitalisation, the patient was able to mobilise independently and required carers to support her four times daily at home. The patient was receiving long-acting injectable antipsychotic treatment for schizophrenia. They were initiated on paliperidone palmitate 150 mg intramuscularly monthly, which was tolerated well. After the fourth dose of the monthly formulation, it was decided to switch the patient to the three-monthly depot formulation: paliperidone palmitate 525 mg intramuscularly, of which the patient was administered two doses three months apart. 

Two months after the last dose, the patient was admitted with severe hypotension (blood pressure - 80/40 mmHg), profound hypothermia (core temperature 29°C), bradycardia (heart rate -48 bpm), and a reduced Glasgow Coma Scale (GCS) of 7/15 (E1V1M5). They received extensive resuscitation, including bag-valve-mask ventilation, warm fluids, and a bear hugger, which resulted in rapid stabilisation and improvement of GCS to 14 (E4V4M6). Initial investigations and clinical assessment indicated multi-organ dysfunction. They were subsequently transferred to the Medical Emergency Care Unit (MECU) and commenced on a metaraminol infusion for circulatory support. Arterial blood gases confirmed decompensated type 2 respiratory failure, and non-invasive ventilation (NIV) was initiated (Table 1*)*. A temporary cessation of metaraminol led to a drop in blood pressure, necessitating reinitiation along with intravenous fluid support.

**Table 1:** Biochemical markers and arterial blood gas sampling results at presentation eGFR: estimated glomerular filtration rate; CRP: C-reactive protein; pO₂: partial pressure of oxygen; pCO₂: partial pressure of carbon dioxide; HCO₃⁻: bicarbonate; TSH: thyroid-stimulating hormone; T₃, T₄: free thyroid hormones; NT-proBNP: N-terminal pro–B-type natriuretic peptide.

Biochemical marker	At presentation	Normal range
Sodium	124 mmol/L	135-145 mmol/L
Potassium	4.9 mmol/L	3.5-5 mmol/L
Magnisium	0.9 mmol/L	0.7- 1.0 mmol/L
Adjusted Calcium	2.05 mmol/L	2.20 – 2.60 mmol/L
Urea	28.6 mmol/L	2.5 – 7.8 mmol/L
Creatinine	441 µmol/L	60-110 µmol/L
eGFR	9mL/min/1.73 m²	>90 mL/min/1.73 m²
CRP	8mg/L	<5mg/L
Heamoglobin	67g/L	115-165g/L
White blood cells	4.5×10⁹/L	4.0 – 11.0 ×10⁹/L
TSH	4.79 mU/L	0.4-5.0 mU/L
Free T4	12.1 pmol/L	pmol/L
Free T3	2.4 pmol/L	pmol/L
Cortisol (9 am)	277 nmol/L	140 – 690 nmol/L
NT-proBNP	2718 pg/ml	< 400 pg/ml
Lactate	< 1.0 mmol/L	0.5 – 2.2 mmol/L
Arterial blood gas		
pH	7.286	7.35 – 7.45
pO2	8.6kPa	10.0 – 13.0 kPa
pCO2	7.57kPa	4.7 – 6.0 kPa
HCO3-	26.5 mmol/L	22 – 28 mmol/L
Base Excess	-0.3 mmol/L	–2 to +2 mmol/L

The patient became oliguric, which did not respond to the trial of intravenous furosemide, and had to be admitted to the Intensive Therapy Unit (ITU) the following day due to worsening fluid overload, hypotension, and deteriorating renal function. The clinical picture was consistent with acute decompensated heart failure with acute-on-chronic renal failure. The patient was commenced on continuous venovenous hemofiltration (CVVH) at 100 mL/h, along with nasogastric feeding and bilevel positive airway pressure (BiPAP), which led to significant cardiovascular and respiratory improvement. Heart rate normalised by the next day, and vasopressor support was successfully weaned. Oral antihypertensives were cautiously reintroduced subsequently.

The patient remained hemodynamically stable through the next three days, and intermittent haemodialysis was initiated. Subsequently, the patient was stepped down to the renal ward for the management of CKD, and an AV fistula was put in place for long-term dialysis.

It was highlighted that due to the CKD, the patient might have an increased level of paliperidone, hence serum concentration was measured earlier during admission, prior to dialysis, and was reported as 62.6 ng/mL (therapeutic range: 20-60 ng/mL). 

Notably, the patient had a similar presentation one month before this episode and required critical care. The event was also characterised by reduced GCS, shortness of breath, dysphagia, and vocal changes lasting three weeks and required respiratory and vasopressor support (metaraminol). They were extensively investigated for the causes, which included a flexible nasal endoscopy, which was unremarkable, and a small right-sided pleural effusion, which was managed conservatively; however, no clear aetiology could be identified. 

**Figure 1:**
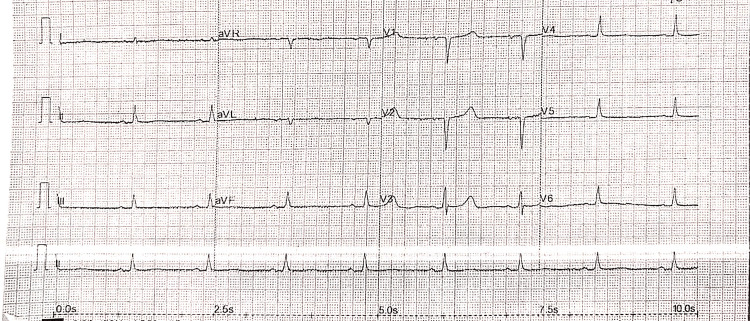
ECG at the time of presentation, demonstrating sinus bradycardia and QTc prolongation ECG: electrocardiogram; QTc: corrected QT interval

A CT head was done, which ruled out acute intracranial events. The electrocardiogram (ECG) revealed sinus bradycardia and a prolonged corrected QT Interval (QTc) of 494 milliseconds (Figure 1)*. *Echocardiography (ECHO) was suggestive of impaired left ventricular (LV) diastolic function with elevated filling pressures.* *The biochemical markers, including estimated glomerular filtration rate (eGFR), C-reactive protein (CRP), and arterial blood gas sampling at the time of admission, are described in Table 1*. *Blood cultures were also done to rule out sepsis, which were reported as no growth. Serum hyponatremia of 124 mmol/L and plasma glucose of 18.5 mmol/L were noted and normalised with supportive management within the next 12 hours.

## Discussion

This case highlights the potential for paliperidone palmitate accumulation and resultant autonomic instability in a patient with chronic kidney disease (CKD), emphasising the critical role of renal function in the pharmacokinetics of long-acting injectable antipsychotics (LAIs) and the need for careful therapeutic monitoring.

Paliperidone undergoes minimal hepatic metabolism [1,2], and kidneys serve the major role in its elimination [1,4] as evidenced by the fact that 59% of the unchanged drug is excreted in the urine within one week after a single 1 mg dose of the immediate-release formulation [1]. One study showed that paliperidone palmitate may remain in the body for at least 5 months, and the half-life of paliperidone palmitate ranges from 25 days to 50 days [9]. Consequently, renal impairment significantly reduces clearance, leading to prolonged half-life and elevated serum concentrations. This is particularly important as the patient had a background of stage 5 chronic renal disease.

Systematic studies are limited for paliperidone dosing in patients with renal failure [1]. In one study, in individuals with impaired renal function, total clearance of paliperidone was found to be reduced by an average of 32% in mild (CrCl = 50 to <80 mL/min), 64% in moderate (CrCl = 30 to <50 mL/min), and 71% in severe (CrCl = 10 to <30 mL/min) kidney impairment. This reduction in creatinine clearance (CrCL) corresponds to an average increase in drug exposure (Area Under the Curve to infinity (AUCinf)) of 1.5-fold, 2.6-fold, and 4.8-fold, respectively, compared to healthy individuals[1]. It is not recommended to administer paliperidone in individuals with moderate to severe renal impairment (creatinine clearance <50 mL/min). For patients with mild renal impairment, with creatinine clearance between 50 and 80 mL/min, a dose reduction is advised [1,6,10].

In our case, the patient’s CrCl was 12 mL/min and eGFR was 9 mL/min/1.73 m2.

Autonomic dysregulation arises from a combination of dopamine D2 and alpha-1 adrenergic receptor blockade. Central D2 antagonism impairs hypothalamic control of temperature and consciousness, leading to hypothermia and reduced GCS as in the current case, while peripheral D2 and alpha-1 blockade result in vasodilation, hypotension, and bradycardia. Additionally, H1 histamine receptor antagonism contributes to sedation and further disrupts thermoregulation. These effects, compounded by impaired perfusion, can culminate in multi-organ dysfunction due to systemic hypoperfusion and central autonomic failure. 

This presentation follows standard dosing of paliperidone palmitate in a patient with chronic kidney disease, which led to a cumulative serum paliperidone concentration of 62.6 ng/mL, slightly above the reported therapeutic range for long-acting injectable formulations (20-60 ng/mL) according to the currently available literature. However, the relationship between paliperidone plasma concentration, clinical response, and adverse effects is not strictly linear [11,12].

This case was further complicated by the patient’s multiple comorbidities, including diabetes, hypertension, prior cerebrovascular disease, and obstructive sleep apnoea, all of which may have contributed to the severity of symptoms. However, the association between paliperidone palmitate dosing followed by subsequent admissions and improvement upon supportive therapy and renal replacement supports likely pharmacotoxicity.

The decision to initiate paliperidone palmitate, a long-acting injectable, in this patient appears to have been made despite the known renal contraindication; this underscores the importance of medication reconciliation and close therapeutic monitoring. This case highlights that in patients with CKD, clinicians should strongly consider non-depot alternatives, more easily titratable oral antipsychotics, or depot formulations with alternative metabolic pathways. Additionally, this case raises an important point regarding the monitoring of serum paliperidone levels, which may offer diagnostic value in complex clinical scenarios, although such testing is not routinely performed in practice due to limited availability and delayed turnaround.

## Conclusions

This case highlights the need for close clinical and therapeutic monitoring when prescribing long-acting antipsychotics in patients with impaired renal function. Paliperidone is a long-acting antipsychotic that antagonises dopamine, serotonin, adrenergic, and histamine receptors. It is primarily excreted through the kidneys. Hence, patients with renal impairment may have reduced clearance of the drug, resulting in autonomic dysregulation secondary to the prolonged blockage of the receptors. This manifests as hypotension, bradycardia, sedation, and disrupted thermoregulation. Patients with a creatinine clearance <50 ml/min should not receive paliperidone, whereas those with a creatinine clearance between 50 and 80 ml/min should have a dose reduction. The monitoring of drug levels in patients with renal impairment receiving long-acting antipsychotics is crucial, and clinicians must carefully assess renal function before initiating or adjusting long-acting antipsychotics and consider alternative treatment strategies in high-risk patients.
